# Urine and Plasma Metabolome of Healthy Adults Consuming the DASH (Dietary Approaches to Stop Hypertension) Diet: A Randomized Pilot Feeding Study

**DOI:** 10.3390/nu13061768

**Published:** 2021-05-22

**Authors:** Shirin Pourafshar, Mira Nicchitta, Crystal C. Tyson, Laura P. Svetkey, David L. Corcoran, James R. Bain, Michael J. Muehlbauer, Olga Ilkayeva, Thomas M. O’Connell, Pao-Hwa Lin, Julia J. Scialla

**Affiliations:** 1 Department of Medicine, University of Virginia School of Medicine, Charlottesville, VA 22903, USA; Js7rk@hscmail.mcc.virginia.edu; 2 College of William and Mary, Williamsburg, VA 23185, USA; miranicchitta@gmail.com; 3 Department of Medicine, Duke University School of Medicine, Durham, NC 27710, USA; crystal.simpson@duke.edu (C.C.T.); laura.svetkey@duke.edu (L.P.S.); james.bain@duke.edu (J.R.B.); olga.ilkayeva@duke.edu (O.I.); pao.hwa.lin@duke.edu (P.-H.L.); 4 Department of Medicine, Virginia Tech Carilion School of Medicine, Roanoke, VA 24016, USA; 5 Duke Molecular Physiology Institute, School of Medicine, Duke University, Durham, NC 27701, USA; michael.muehlbauer@duke.edu (M.J.M.); thoconne@iu.edu (T.M.O.); 6 Center for Genomics and Computational Biology, School of Medicine, Duke University, Durham, NC 27708, USA; David.corcoran@duke.edu; 7 Department of Otolaryngology, School of Medicine, Indiana University, Indianapolis, IN 46202, USA; 8 Department of Public Health Sciences, School of Medicine, University of Virginia, Charlottesville, VA 22903, USA

**Keywords:** nutrition, polyphenols, blood pressure, DASH, metabolomics

## Abstract

We aimed to identify plasma and urine metabolites altered by the Dietary Approaches to Stop Hypertension (DASH) diet in a post-hoc analysis of a pilot feeding trial. Twenty adult participants with un-medicated hypertension consumed a Control diet for one week followed by 2 weeks of random assignment to either Control or DASH diet. Non-missing fasting plasma (n = 56) and 24-h urine (n = 40) were used to profile metabolites using untargeted gas chromatography/mass spectrometry. Linear models were used to compare metabolite levels between the groups. In urine, 19 identifiable untargeted metabolites differed between groups at *p* < 0.05. These included a variety of phenolic acids and their microbial metabolites that were higher during the DASH diet, with many at false discovery rate (FDR) adjusted *p* < 0.2. In plasma, eight identifiable untargeted metabolites were different at *p* < 0.05, but only gamma-tocopherol was significantly lower on DASH at FDR adjusted *p* < 0.2. The results provide insights into the mechanisms of benefit of the DASH diet.

## 1. Introduction

The Dietary Approaches to Stop Hypertension (DASH) diet emphasizes consumption of fruits, vegetables, low-fat dairy, whole grains, fish, poultry, and nuts, while limiting red meat, sweets, and sugar-containing beverages [1]. In randomized clinical trials, DASH reduces systolic blood pressure (BP) by an average of 5.2 mmHg over durations of 2–24 weeks [2]. Observational studies suggest that DASH may also reduce the risks of atherosclerotic cardiovascular disease, stroke, diabetes, and kidney disease [3,4]. However, the mechanism of benefit of the DASH diet is poorly understood. Metabolomics phenotyping can help characterize the changes in body chemistry following a dietary intervention, such as DASH, and thus identify potential mechanisms of benefit [5]. Understanding the potential mechanisms of DASH using informative biomarkers could help to fine-tune implementation strategies. Thus, in this post-hoc analysis, we characterized changes in the plasma and urine metabolome among generally healthy patients with un-medicated stage 1 hypertension while consuming the DASH diet.

## 2. Materials and Methods

**Figure 1:**
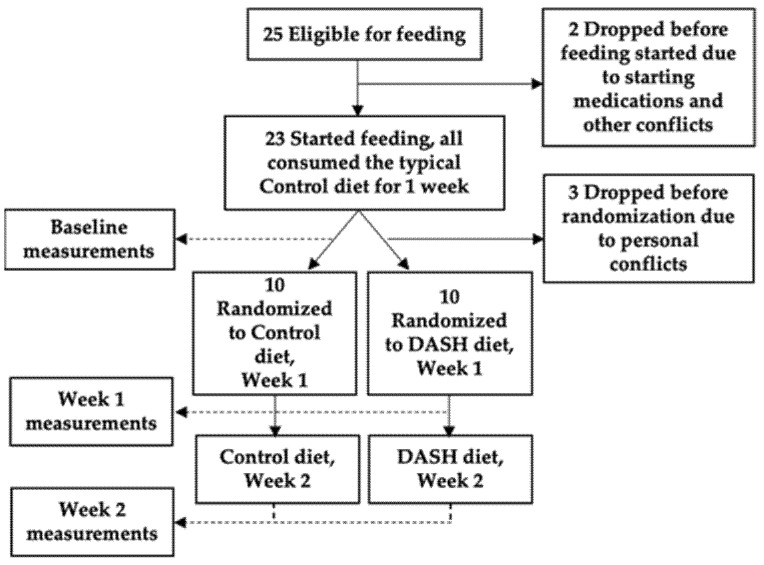
Study flow chart. Adapted from original published manuscript [6].

The DASH Mechanism Study (n = 20) was a single-center, 3-week controlled feeding randomized clinical trial designed to evaluate the mechanism of DASH as previously published [6]. All participants were 22 years of age or older, met the Joint National Commission (JNC) 7 criteria for stage 1 hypertension (systolic BP of 140–159 or diastolic BP of 90–99 mmHg) [7], and were not taking any anti-hypertensive therapies. Participants were excluded if they had a glomerular filtration rate (GFR) < 60 mL/min/1.73 m^2^, diabetes, active heart disease, or any other serious medical conditions. After screening, confirmation of eligibility and informed consent, all participants first consumed a Control diet representative of typical American intake for one week. Baseline sample collections including fasting plasma and 24-h urine were obtained at the end of this period as a baseline assessment. Immediately after the completion of the baseline period, participants were randomized 1:1 to 2 additional weeks of either the Control diet or the DASH diet with sample data collection at the end of each week (randomized period). Figure 1 shows the study flow chart. All recruitment occurred between 2007 and 2009 at Duke University and samples were stored at −80 °C until metabolomics testing in 2014.

Adherence to the study diet was monitored daily at on-site feeding visits and with diet logs. When compared to the Control diet, the DASH diet is designed to be lower in total fat and saturated fat, but higher in both mono- and polyunsaturated fats. DASH is also higher in protein, fiber, potassium, magnesium, and calcium content. Although the original DASH diet was designed with the specific nutrient targets, these were later translated into food groups for practical implementation. Compared to the Control diet, the DASH diet is rich in fruits, vegetables, low-fat dairy, nuts and seeds, and lower in red meats, sweets and sugar-sweetened beverages.

To characterize the metabolic impacts of the DASH diet, we performed untargeted gas chromatography/mass spectrometry (GC/MS) on stored non-missing fasting plasma (n = 56 out of 60 time points were available) and 24-h urine, diluted in pure water to achieve constant creatinine concentration across samples (n = 40 out of 60 time points were available). Detailed GC/MS methods are published elsewhere [8]. All metabolites are quantified as the log_2_ peak areas. Within each untargeted dataset, metabolites were filtered out if they were detected in <50% of samples. Remaining untargeted data was imputed using k-nearest neighbor (k = 6). A generalized estimating equation model was implemented in R for each metabolite to compare levels in the DASH versus Control during the randomized weeks accounting for correlation of repeated measures within the same individual. The Benjamini-Hochberg false discovery rate (FDR) threshold <0.2 was used to account for multiple testing, while allowing discovery in this exploratory analysis. Metabolites with an unknown Chemical Abstracts Services number were not reported.

Analyses were performed following the initial Control diet period to evaluate any baseline imbalance between the two randomized groups. This was followed by models for the 2 randomized feeding weeks for the difference between individuals consuming DASH and Control. We also performed sensitivity analyses in R using linear mixed models to determine the within-subject change for each metabolite between baseline and the first randomized week, as well as between baseline and the second randomized week, focusing interpretation on metabolites identified in the primary approach. These change models were not selected as the primary analysis because of missing samples, which resulted in loss of participants from the model and reduced power.

## 3. Results

Overall, 20 participants completed the DASH Mechanism Study, as previously reported [6]. The average age range in participants of DASH Mechanism was 46.1 ± 9.0 years. The population included 13 females and had an average body mass index (BMI) of 33.9 ± 6.6 kg/m^2^ and average systolic BP of 144.2 ± 9.4 mmHg at screening. Table 1 shows baseline participant characteristics by randomized diet groups. Clinical parameters including changes in blood pressure, endothelial function, pulse wave velocity, and plasma nitric oxide levels were reported in the original study. Consumption of DASH diet resulted in significant reductions of blood pressure starting at the end of week one (both systolic and diastolic) and end of week two (systolic) [6].

**Figure 2:**
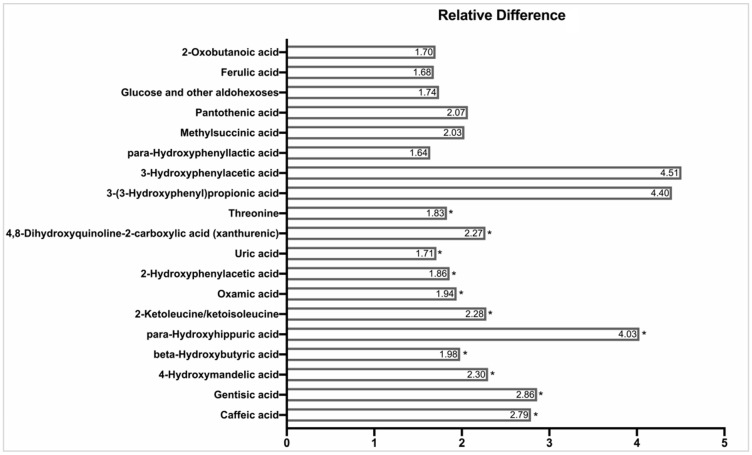
Relative Difference in Nominally Significant Metabolites from Untargeted Urine GC/MS during Randomized DASH and Control Feeding Periods. Relative difference represents the ratio of peak areas by GC/MS. Differences are tested using generalized estimating equation models evaluating the difference in log_2_ peak areas in non-missing samples obtained during randomized study weeks on either DASH or Control diet. Relative differences re-express absolute log_2_ difference coefficients (β) as 2^β^. Significance at a false discovery rate (FDR) adjusted *p* < 0.20 is indicated by an asterisk (*). Metabolites are ordered by statistical significance with the most statistically significant metabolites beginning at the bottom near the *x*-axis. Several metabolites had between 10–20 imputed values including caffeic acid, gentisic acid, uric acid and threonine.

We found >150 unique identifiable metabolites in the urine. Five metabolites differed in the two groups at baseline with a nominal *p*-value of <0.05, reflecting modest baseline imbalance in profiles across groups. These metabolites included lower levels of octanoic acid (*p* = 0.02), adipic acid (*p* = 0.02), and cinnamoyl-glycine (*p* = 0.04) and higher levels of methyl-succinic acid (*p* = 0.03) and 2-hydroxyphenylacetic acid (*p* = 0.04). Nineteen urine metabolites differed between Control and DASH with a nominal *p*-value < 0.05 (Figure 2 and Table 2). Eleven of these metabolites had FDR adjusted *p* < 0.20, including caffeic acid, gentisic acid, 4-hydroxymandelic acid, beta-hydroxybutyric acid, para-hydroxy-hippuric acid, keto-leucine/keto-isoleucine, oxamic acid, 2-hydroxyphenylacetic acid, uric acid, 4,8-dihydroxyquinoline-2-carboxylic acid, and threonine.

In plasma, we found >110 uniquely annotated metabolites of known identity. At the end of the first week of Control diet (baseline), 11 metabolites differed in the two groups, reflecting modest baseline imbalance in profiles. Unbalanced metabolites included 2-ketoleucine/keto-isoleucine (*p* = 0.01), beta-hydroxybutyric acid (*p* = 0.02), hydroxyprolines (*p* = 0.02), 2-ketovaline (*p* = 0.02), leucine (*p* = 0.03), valine (*p* = 0.04), oleic acid (*p* = 0.04), and pyruvic acid (*p* = 0.04). We found eight plasma metabolites that differed between Control and DASH during randomized weeks with a nominal *p*-value ≤ 0.05. These metabolites included lower gamma-tocopherol, hydroxyprolines, and methionine, and higher oleic acid, beta-hydroxybutyric acid, myoinositol, citric acid/iso-citric acid, and beta-sitosterol on DASH. We interpret these cautiously as several of these were imbalanced at baseline (hydroxyprolines, oleic acid, beta-hydroxybutyric acid). Only gamma-tocopherol remained different at an FDR adjusted *p* < 0.20, which was not expected given the higher fruit and vegetable composition of DASH.

In sensitivity analyses of within participant change, the two urine metabolites 3-(3-hydroxyphenyl)propionic acid and threonine increased more in DASH vs. Control at the end of the first randomized week (*p* < 0.05 and FDR-adjusted *p* < 0.2). No metabolites in plasma or urine changed at both a nominal and FDR-adjusted *p*-value between baseline and week 2, but power was limited in all analyses due to missing data.

## 4. Discussion

In this study, we used metabolomics to examine changes that occur with the consumption of the DASH diet in comparison to a typical American diet. Metabolomics can provide us with a ‘read out’ of the metabolic impact of DASH and identify potential objective biomarkers which could be used to further study and monitor favorable responses to DASH. In summary, we found several urine metabolites that were higher in DASH vs. Control participants during randomized feeding weeks. These primarily consisted of small organic acids of a variety of classes including phenols/polyphenols, carboxylic acids, and ketoacids, among others.

Phenols and their derivatives have antioxidant and anti-inflammatory properties [9,10,11] with potential BP lowering effects through mechanisms such as scavenging reactive oxygen species in the vasculature, inhibiting vascular smooth muscle cell proliferation, and enhancing nitric oxide (NO) availability [12]. Caffeic acid, ferulic acid and other related metabolites are known to be absorbed in the gastrointestinal tract and are abundant in coffee and tea [13,14,15]. While individuals in our study were allowed to consume coffee and tea up to a combination of three servings a day, it is unlikely that their intake would have differed significantly between Control and DASH to result in the observed differences in the metabolites. Thus, it is more likely that the high caffeic and ferulic acid content of fruits and vegetables is responsible for the noted differences in excretion [16]. Many of the phenolic acids identified are also products of microbial phenol catabolism, including 3-(3-hydroxyphenyl) propionic acid, 2-hydroxyphenylacetic acid, para-hydroxy-hippuric acid, and were elevated on DASH [17]. Although we did not directly characterize the microbiota, the higher excretion of microbial metabolites may suggest an impact of DASH on the microbiota itself as a mechanism of benefit.

We identified gentisic acid which is a metabolite of salicylic acid or aspirin. Although aspirin intake is a common source of these metabolites, in the context of this feeding study, this finding could be due to the abundance of salicylic acid and related botanical phenolic compounds in fruits and vegetables [18,19]. Although research on dietary salicylates is limited, aspirin and sodium salicylate have been shown to raise inducible NO synthase (iNOS) and enhance NO production [20,21]. While acetylsalicylate (aspirin) is not found in foods, the similar efficacy of aspirin and sodium salicylate indicate that the acetyl moiety is not a significant factor in the effect on NO [21,22]. Improvement in NO bioavailability was a candidate mechanism of action of DASH identified by the primary results of the DASH Mechanism Study [6]. Further studies are needed to understand if salicylates in foods contribute to this effect.

The results of our study are consistent with prior research. In another recent small study by Reisdorph et al. (n = 19), investigators evaluated metabolic profiles of 12 foods common in DASH-style diets. They evaluated urine LC-MS metabolomics and detected many of these food specific metabolites in urine samples from participants consuming the DASH diet, particularly urine phenolic compounds from fruits and vegetables [23]. This is consistent with our finding of a higher urine phenolic acids in the DASH group which contains higher levels of fruits and vegetables. Rebholz et al. evaluated serum metabolites using a combination of GC-MS and LC-MS in stored samples from the original DASH trial [24]. With a much larger sample size, they identified 96 serum metabolites more common in DASH. One of the top hits, chiro-inositol, was from the same chemical class as myoinositol, which was found in our plasma samples. Inositol can be found in both animal and plant cells with its derivatives most abundant in grains, beans, nuts, seeds, and fruits [24,25]. These observations are consistent with the high content of fruits, nuts, and seeds in the DASH diet.

Our study has a few limitations. Due to the small sample size, we could not adjust for baseline values and other clinical covariates in this hypothesis-generating analysis. Thus, it is possible that some of the differences we noted were chance differences in groups at baseline and effects of missing samples. We primarily utilized semi-quantitative, untargeted tools for metabolomics, which are an initial discovery step that needs further refinement before clinical application. Given that most of the urinary metabolites we observed belong to the class of phenolic acids, which may have antioxidant activities, future studies should also assess total antioxidant activity to further understand the mechanisms of both urinary and plasma metabolites of DASH diet. This study also has multiple strengths. According to a recent review by Kim et al. [26], there are about 17 studies which have investigated the metabolomics signatures of different dietary patterns, including the DASH diet. However, out of those studies, 12 have used cross-sectional designs, three have used data from behavioral intervention trials, and only two studies are controlled feeding trials. In the current study, we used a highly-controlled feeding protocol to ensure optimal delivery of the DASH diet and we investigated metabolites related to a dietary pattern rather than a single food or nutrient.

## 5. Conclusions

The results of this study demonstrate several metabolite differences comparing DASH to the typical American diet. Consumption of the DASH diet may increase urine levels of a set of bioactive phenolic compounds (e.g., caffeic acid) and their metabolites formed by the gastrointestinal microbiota (e.g., 3-(3-hydroxyphenyl) propionic acid; 2-hydroxyphenylacetic acid). The results of our study, along with results of more follow up studies, can be useful in understanding potential mechanisms of action of the DASH diet.

## Figures and Tables

**Table 1:** Baseline participant characteristics by randomized diet groups.

Characteristic (Mean ± SD or n (%))	DASH Diet	Control Diet
N	10	10
Age (years)	46.1 ± 9.0	42.4 ± 6.3
Female Sex (%)	7 (70.0)	6 (60.0)
Race		
African American (%)	6 (60.0)	8 (80.0)
Caucasian (%)	3 (30.0)	2 (20.0)
Other (%)	1 (10.0)	0 (0.0)
Body Weight (kg)	85.0 ± 15.5	107.0 ± 18.5
Body Mass Index (kg/m^2^)	31.0 ± 5.9	36.9 ± 6.1
Blood Pressure (BP)		
Systolic BP (mmHg)	142.8 ± 7.03	145.6 ± 11.5
Diastolic BP (mmHg)	88.8 ± 6.67	88.2 ± 5.68

**Table 2:** Annotation of identified urine metabolites as candidates modified by DASH.

Class	Metabolite	Sub-Class *	Example Sources *
Phenolic Acids	^¥^ Caffeic acid	Hydroxycinnamic acids	Wine; whole grains; herbs; fruits; vegetables
	Ferulic acid	Hydroxycinnamic acids	Whole grains; cocoa; fruits; vegetables; herbs
	^¥^ 4-Hydroxymandelic acid	Hydroxyphenylacetic acids	Microbial phenol/polyphenol metabolite
	3-Hydroxyphenylacetic acid	Hydroxyphenylacetic acids	Microbial phenol/polyphenol metabolite
	^¥^ 2-Hydroxyphenylacetic acid	Hydroxyphenylacetic acids	Microbial phenol/polyphenol metabolite
	^¥^ Para-Hydroxyhippuric acid	Hydroxybenzoic acids	Microbial phenol/polyphenol metabolite
	^¥^ Gentisic Acid	Hydroxybenzoic acids	Aspirin; selected fruits; herbs
	3-(3-Hydroxyphenyl)propionic acid	Hydroxyphenylpropanoic acid	Microbial phenol/polyphenol metabolite
	Para-Hydroxyphenyllactic acid	Hydroxyphenylpropanoic acid	Microbial phenol/polyphenol metabolite
Quinolines	^¥^ 4,8-Dihydroxyquinoline-2-carboxylic acid (xanthurenic acid)	Quinoline carboxylic acids	Metabolite of tryptophan, protein sources
Keto Acids and Derivatives	^¥^ 2-Ketoleucine	Short-chain keto acids/ derivatives	Branched chain amino acid degradation
	2-Oxobutanoic acid	Short-chain keto acids/ derivatives	Amino acid degradation
Fatty acyls	Methylsuccinic acid	Fatty acids and conjugates	Involved in isoleucine metabolism
Imidazopyrimidines	^¥^ Uric acid	Purines and purine derivatives	Final product of purine metabolism; Protein sources
Organooxygen Compounds	Pantothenic acid (vitamin B5)	Alcohols and polyols	Whole grain; legumes; eggs; meat
	Glucose and other aldohexoses	Carbohydrates	Many
Carboxylic Acids and Derivatives	^¥^ Oxamic acid	Carboxylic acid derivatives	Amine derivative of oxalate
Hydroxy Acid and Derivatives	^¥^ β-Hydroxybutyric acid	β-Hydroxy acids	Ketone body
Amino Acids	^¥^ Threonine	NA	Cottage cheese; poultry; fish; meat; lentils; black bean; sesame seeds

* Sources: Human Metabolome Database (hmdb.ca), U.S. National Library of Medicine (www.pubchem.ncbi.nlm.nih.gov; access date: 18 November 2019), National Institute of Standards and Technology (webbook.nist.gov; access date: 18 November 2019), and Phenol-Explorer (www.phenol-explorer.eu; access date: 18 November 2019). ^¥^ Metabolites were identified at nominal *p* < 0.05 and FDR adjusted *p* < 0.20.

## Data Availability

The data presented in this study are available on request from the corresponding author. The data are not publicly available due to ethical and privacy restrictions.
